# Supplementary material for: Chromosome-wide mechanisms to decouple gene expression from gene dose during sex-chromosome evolution
Source: eLife. 2016 Aug 30;5:e17365. doi: 10.7554/eLife.17365 (PMC5047749; doi:10.7554/eLife.17365)
Supplement: Supplementary file 1. — DOI: http://dx.doi.org/10.7554/eLife.17365.016 [file elife-17365-supp1.docx]

**Supplementary File 1. Strains used in this study**

| **Strain** | **Genotype** | **Chr.** | **Insert site (bp)** |
| --- | --- | --- | --- |
|  |  |  |  |
| EG6173 | *oxSi259[Peft-3:GFP cbr-unc-119*(+)*] I; unc-119(ed3) III* | I | 2851041 |
| EG6129 | *oxSi239[Peft-3:GFP rex-32 cbr-unc-119*(+*)] I; unc-119(ed3) III* | I | 2851041 |
| TY5726 | *oxSi239[Peft-3:GFP rex-32 cbr-unc-119*(+*)] I; rex-32(y575) X* | I | 2851041 |
| EG6397 | *oxSi342[Peft-4:GFP cbr-unc-119*(+)*] I; unc-119(ed3) III* | I | 2851041 |
| EG6810 | *oxTi134[Pdpy-30:GFP:H2B cbr-unc-119*(+)*] I; unc-119(ed3) III* | I | 6294488 |
| EG6171 | *oxSi257[Peft-3:GFP cbr-unc-119*(+)*] I; unc-119(ed3) III* | I | 11269576 |
| EG6133 | *oxSi243[Peft-3:GFP rex-32 cbr-unc-119*(+)*] I; unc-119(ed3) III* | I | 11269576 |
| EG6487 | *oxSi363[Peft-4:GFP cbr-unc-119*(+)*] I; unc-119(ed3) III* | I | 11269576 |
| EG6070 | *oxSi221[Peft-3:GFP cbr-unc-119*(+)*] II; unc-119(ed3) III* | II | 8420158 |
| EG6074 | *oxSi225[Peft-3:GFP rex-32 cbr-unc-119*(+)*] II; unc-119(ed3) III* | II | 8420158 |
| EG6398 | *oxSi343[Peft-4:GFP cbr-unc-119*(+)*] II; unc-119(ed3) III* | II | 8420158 |
| EG6826 | *unc-119(ed3) oxTi150[Pdpy-30:GFP:H2B cbr-unc-119*(+)*] III* | III | 11558130 |
| EG6800 | *unc-119(ed3) III; oxTi124[Pdpy-30:GFP:H2B cbr-unc-119*(+)*] IV* | IV | 54707 |
| EG6401 | *unc-119(ed3) III; oxSi346[Peft-3:GFP cbr-unc-119*(+)*] IV* | IV | 5014697 |
| EG6402 | *unc-119(ed3) III; oxSi347[Peft-3:GFP cbr-unc-119*(+)*] IV* | IV | 5014697 |
| EG6135 | *unc-119(ed3) III; oxSi245[Peft-3:GFP rex-32 cbr-unc-119*(+)*] IV* | IV | 5014697 |
| EG6136 | *unc-119(ed3) III; oxSi246[Peft-3:GFP rex-32 cbr-unc-119*(+)*] IV* | IV | 5014697 |
| EG6403 | *unc-119(ed3) III; oxSi348[Peft-4:GFP cbr-unc-119*(+)*] IV* | IV | 5014697 |
| EG6831 | *unc-119(ed3) III; oxTi155[Pdpy-30:GFP:H2B cbr-unc-119*(+)*] IV* | IV | 9500806 |
| EG7942 | *unc-119(ed3) III; oxTi393[Peft-3:tdTomato:H2B:unc-54UTR cbr-unc-119*(+)*] V* | V | 4748478 |
| TY5758 | *unc-119(ed3) III; oxTi387[Peft-3:tdTomato:H2B:unc-54UTR cbr-unc-119*(+)*] V* | V | 5177680 |
| EG7565 | *unc-119(ed3) III; oxTi392[Peft-3:tdTomato:H2B:unc-54UTR cbr-unc-119*(+)*] V* | V | 5573254 |
| EG7957 | *unc-119(ed3) III; oxTi389[Peft-3:tdTomato:H2B:unc-54UTR cbr-unc-119*(+)*] V* | V | 14015406 |
| EG7960 | *unc-119(ed3) III; oxTi401[Peft-3:tdTomato:H2B:unc-54UTR cbr-unc-119*(+)*] V* | V | 15894041 |
| EG7827 | *unc-119(ed3) III; oxTi309[Peft-3:tdTomato:H2B:unc-54UTR cbr-unc-119*(+)*] V* | V | 20158479 |
| TY5757 | *unc-119(ed3) III; oxTi316[Peft-3:tdTomato:H2B:unc-54UTR cbr-unc-119*(+)*] X* | X | 1794165 |
| EG7977 | *unc-119(ed3) III; oxTi410[Peft-3:tdTomato:H2B:unc-54UTR cbr-unc-119*(+)*] X* | X | 2563231 |
| EG6413 | *unc-119(ed3) III; oxSi352[Peft-3:GFP cbr-unc-119*(+)*] X* | X | 4347080 |
| EG6415 | *unc-119(ed3) III; oxSi354[Peft-3:GFP rex-32 cbr-unc-119*(+)*] X* | X | 4347080 |
| EG6416 | *unc-119(ed3) III; oxSi355[Peft-3:GFP rex-32 cbr-unc-119*(+)*] X* | X | 4347080 |
| EG6489 | *unc-119(ed3) III; oxSi365[Peft-4:GFP cbr-unc-119*(+)*] X* | X | 4347080 |
| EG7215 | *unc-119(ed3) III; oxTi334[Peft-3:tdTomato:H2B:unc-54UTR cbr-unc-119*(+)*] X; gaIs283.* | X | 4348096 |
| EG6807 | *unc-119(ed3) III; oxTi131[Pdpy-30:GFP:H2B cbr-unc-119*(+)*] X* | X | 9575893 |
| EG7990 | *unc-119(ed3) III; oxTi400[Peft-3:tdTomato:H2B:unc-54UTR cbr-unc-119*(+)*] X* | X | 9672421 |
| EG6806 | *unc-119(ed3) III; oxTi130[Pdpy-30:GFP:H2B cbr-unc-119*(+)*] X* | X | 10172711 |
| EG7993 | *unc-119(ed3) III; oxTi412[Peft-3:tdTomato:H2B:unc-54UTR cbr-unc-119*(+)*] X* | X | 11048713 |
| EG6801 | *unc-119(ed3) III; oxTi125[Pdpy-30:GFP:H2B cbr-unc-119*(+)*] X* | X | 12313579 |
| EG7994 | *unc-119(ed3) III; oxTi395[Peft-3:tdTomato:H2B:unc-54UTR cbr-unc-119*(+)*] X* | X | 13482858 |
| EG6110 | *unc-119(ed3) III; oxSi231[Peft-3:GFP cbr-unc-119*(+)*] X* | X | 15574710 |
| EG6111 | *unc-119(ed3) III; oxSi232[Peft-3:GFP cbr-unc-119*(+)*] X* | X | 15574710 |
| EG6076 | *unc-119(ed3) III; oxSi227[Peft-3:GFP rex-32 cbr-unc-119*(+)*] X* | X | 15574710 |
| EG6078 | *unc-119(ed3) III; oxSi229[Peft-3:GFP rex-32 cbr-unc-119*(+)*] X* | X | 15574710 |
| EG6411 | *unc-119(ed3) III; oxSi350[Peft-4:GFP cbr-unc-119*(+)*] X* | X | 15574710 |
| EG6834 | *unc-119(ed3) III; oxTi158[Pdpy-30:GFP:H2B cbr-unc-119*(+)*] X* | X | 16626759 |
