# Supplementary material for: Chromosome-wide mechanisms to decouple gene expression from gene dose during sex-chromosome evolution
Source: eLife. 2016 Aug 30;5:e17365. doi: 10.7554/eLife.17365 (PMC5047749; doi:10.7554/eLife.17365)
Supplement: Supplementary file 2. — DOI: http://dx.doi.org/10.7554/eLife.17365.017 [file elife-17365-supp2.docx]

**Supplementary File 2. Oligos used in this study**

| **Name** | **Sequence** | **Target** | **Purpose** |
| --- | --- | --- | --- |
|  |  |  |  |
| BWP26F | CTGCTGGACAGGAAGATTACG | *cdc-42* | qRT-PCR (normalization) |
| BWP26R | CTCGGACATTCTCGAATGAAG |  |  |
| BWP47F | GGAGGAAGAGGTGGATTCGG | *F41E7.5* | qRT-PCR |
| BWP47R | TTCCACGGATGATGGTCTCC |  |  |
| BWP49F | CCTGAGATCGATGCTGTGCT | *F47B10.2* | qRT-PCR |
| BWP49R | AGCTCCTCCATAGCCTCCAA |  |  |
| BWP52F | AACTCTCAACACCGTGGACC | *F19F10.9* | qRT-PCR |
| BWP52R | TTTGGCATCCATTTCACGGC |  |  |
| BWP54F | ACCCACGACATTGCTCTTGT | *H06O01.1.1* | qRT-PCR & ChIP (normalization) |
| BWP54R | AGTTTTGGGGCAGCTCTCTC |  |  |
| BWP55F | GAAAGCGCCCAAACTGGAAG | *Y38A10A.5* | qRT-PCR & ChIP (normalization) |
| BWP55R | ATCCCAGTCCTCTGGCTTCT |  |  |
| BWP15F | GGCAGACAAACAAAAGAATGG | *gfp* | qRT-PCR & ChIP |
| BWP15R | GGACAGGTAATGGTTGTCTGG |  |  |
| BWP60F | TTCTTATGCTCCCCATCTGC | *cbr-unc-119* | qRT-PCR & ChIP |
| BWP60R | TCTCGAAAAGCACTTGCTCA |  |  |
| BWP65F | ACCTGGTGGAGTTCAAGACC | *tdTomato* | qRT-PCR |
| BWP65R | GAGGTGATGTCCAGCTTGGT |  |  |
| BWP95F | CGTATGTCCCAAAATCAATCTG | *C15C7.5* | qRT-PCR |
| BWP95R | TTATTTTTCGGTGCGTTTGA |  |  |
| BWP88F | GCGTGCGTACAAAAGGAGAC | *rex-1* | ChIP |
| BWP88R | TCTTTCCCCTGCCCAATTAC |  |  |
| BWP90F | CACTCCCCAGCTAATTTGGA | *rex-32* (center) | ChIP |
| BWP90R | TTCCCTTGTTGCGGAGATAG |  |  |
| BWP73F | AGGGGTCGGTTGAGTGAAT | *rex-32* (junction in transgene) | ChIP |
| BWP73R | GGGTTAATACGACTCACTAGATGC |  |  |
| BWP74F | TTGGAAAAGCAGTCATTTCG | no DCC region on *X*  (X:15580560-15580636) | ChIP |
| BWP74R | AGAAACGCCGATGTTGTTGT |  |  |
| BWP89F | GACCCCTGAAGTGTTTTCCA | *rex-32* (endogenous junction) | ChIP |
| BWP89R | TGACCAGCTGATATTCAGTAGAAC |  |  |
|  |  |  |  |
